# A randomized controlled trial of cognitive remediation and d-cycloserine for individuals with bipolar disorder

**DOI:** 10.1186/s40359-014-0041-4

**Published:** 2014-10-10

**Authors:** Nicholas JK Breitborde, Spencer C Dawson, Cindy Woolverton, David Dawley, Emily K Bell, Kaila Norman, Angelina Polsinelli, Beth Bernstein, Pamela Mirsky, Christine Pletkova, Felix Grucci, Carly Montoya, Bernard Nanadiego, Ehsan Sarabi, Michael DePalma, Francisco Moreno

**Affiliations:** Department of Psychiatry, The University of Arizona, Tucson, AZ USA; Department of Psychology, The University of Arizona, Tucson, AZ USA

## Abstract

**Background:**

Cognitive remediation (CR) has shown significant promise in addressing the cognitive deficits that accompany serious mental illness. However, this intervention does not appear to completely ameliorate the cognitive deficits that accompany these illnesses. D-cycloserine (DCS), an NMDA receptor partial agonist, has been shown to enhance the therapeutic benefits of learning-based psychosocial interventions for psychiatric disorders. Thus, the goal of this study is to examine the utility of combining cognitive remediation and d-cycloserine in the treatment of cognitive deficits among individuals with bipolar disorder.

**Methods/Design:**

Approximately forty individuals with bipolar disorder will be recruited to participate in this study. Participants will be randomized to one of two study arms: CR + DCS or CR + placebo. The primary outcome for this study is change in cognitive functioning. We will also examine several secondary outcomes, including the rate of change of cognitive functioning, social functioning, and symptomatology.

**Discussion:**

Cognitive deficits are a rate-limiting factor in functional recovery among individuals with bipolar disorder. Unfortunately, treatment options for these deficits are limited. The results of the proposed study may reveal a valuable intervention strategy (i.e., CR with concurrent DCS) to improve cognitive functioning among individuals with bipolar disorder. Ultimately, this treatment strategy may prove useful in addressing the cognitive deficits that are ubiquitous across serious mental illnesses.

**Trial registration:**

ClinicalTrials.gov NCT01934972.

## Background

In the 2013 update to Global Burden of Disease Study, bipolar disorder was identified as one of the top ten most debilitating psychiatric illnesses (Salomon et al. 2013). Although a significant portion of the illness-related disability that accompanies bipolar disorder stems from the episodes of manic and dysthymic mood that define this disorder, there is growing recognition that deficits in cognitive functioning are also significant contributors to the disability experienced by individuals with bipolar disorder. More specifically, individuals with bipolar disorder experience deficits in multiple domains of cognitive functioning that are present throughout the manic, dysthymic, and euthymic phases of the illness (Green 2006). These deficits serve as a rate-limiting factor with regard to many aspects of the recovery process in bipolar disorder, including social and vocational functioning (Bearden et al. 2011; Baune et al. 2013; Dickerson et al. 2004).

Given the nearly ubiquitous occurrence of cognitive deficits among individuals with serious mental illness, there is significant interest in developing interventions designed to improve cognitive functioning. One intervention that has shown great promise in ameliorating these cognitive deficits is cognitive remediation (CR). This intervention, which is recognized as a “best practice” in the treatment of serious mental illness (Browne et al.; APA/CAAP Task Force on Serious Mental Illness and Severe Emotional Disturbance 2007), is typically comprised of a series of repeated exercises delivered by a clinician or via a computer that are designed to improve performance in cognitive functioning. To date, one trial of cognitive remediation among individuals with bipolar disorder has been completed (Deckersbach et al. 2010). In this open trial, Deckersbach and colleagues provided 18 individuals with bipolar disorder with a series of cognitive behavioral therapy sessions in which participants learned strategies to manage and monitor mood, improve planning and organization, and increase attention and memory. At the end of treatment, participants reported improvements in self-rated organization and planning. However, the lack of a control condition and objective measures of cognitive functioning suggest that the promising results of this study should be interpreted with caution. A more recent trial of functional remediation, an intervention designed to address both cognitive and functional deficits (Martínez-Arán et al. 2011), found no effects of this intervention on cognitive functioning among individuals with bipolar disorder (Torrent et al. 2013).

Despite the apparent benefits of CR for individuals with serious mental illnesses, this intervention does not appear to completely ameliorate the cognitive deficits that accompany these illnesses. Consequently, there is growing interest in the use of pharmacological cognitive enhancers to increase the benefits of CR (Goff et al. 2011; Krystal et al. 2009; Chou et al. 2012). In such models, cognitive enhancers are not thought to promote improved cognition by themselves; rather they are hypothesized to augment the physiological mechanism(s) through which CR produces its therapeutic benefits (e.g., learning (Hofmann et al. 2011) or neuroplasticity (Cain et al. 2014)) One such promising cognitive enhancer is d-cycloserine (DCS)—a partial or full agonist of NMDA receptors (depending on the subunit composition of the receptor (Dravid et al. 2010)) that may facilitate the learning process for emotional and non-emotional information through the promotion of long-term potentiation (Assini et al. 2009; Lelong et al. 2001; Onur et al. 2010; Ressler et al. 2004). Of note, dysfunction in glutamate transmission, which is regulated in part by NMDA receptors, has been hypothesized to contribute to the cognitive dysfunction that accompanies bipolar disorder (Goldberg & Roy Chengappa 2009).

To date, several controlled trials have found that DCS augmentation can enhance the therapeutic benefits of learning-based psychosocial interventions for anxiety disorders (Norberg et al. 2008; Bontempo et al. 2012). However, we are unaware of any study that specifically examines the benefits of pairing DCS with CR for individuals with bipolar disorder. One recently completed trial examined whether a weekly dose of 50 mg DCS enhanced the benefits of CR among individuals with schizophrenia (Cain et al. 2014). This study found that subjects who received both DCS and cognitive remediation showed greater improvements in performance on an auditory discrimination task as compared to subjects who received cognitive remediation alone. Conversely, only subjects who received cognitive remediation alone experienced improvements in global cognition as measured using the composite score for the MATRICS Consensus Cognitive Battery (MCCB: (Nuechterlein et al. 2008)). Subjects who received both cognitive remediation and DCS showed no improvement in the composite or specific cognitive domain scores for the MCCB.

Thus, the goal of this study is to complete a controlled trial of CR and DCS among individuals with bipolar disorder. Of note, unlike the previous negative trial of cognitive remediation and DCS in schizophrenia (Cain et al. 2014), our study included a higher dose of DCS (250 mg two times per week versus 50 mg one time per week) and a longer (52 sessions over 26 weeks versus 24–40 sessions over 8 weeks) and more intensive cognitive remediation training program. The results of this study may ultimately inform the provision of care for the cognitive deficits that accompany bipolar disorder.

## Methods/Design

**Figure 1:**
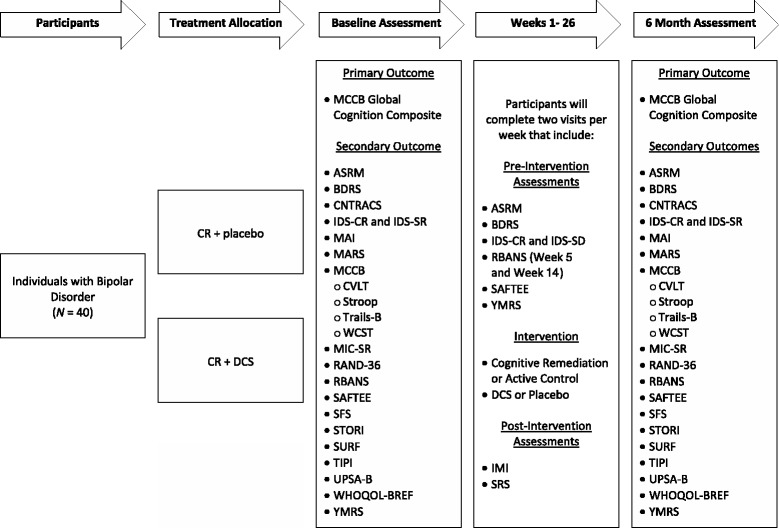
**Participant flow and study design.**

The methods/design of this study are described below and are summarized in Figure 1. This project was approved by the University of Arizona Human Subjects Protection Program and is registered with ClinicalTrials.gov (NCT01934972).

### Participants

Approximately, forty individuals with bipolar disorder will be recruited to participate in this study. Eligibility criteria include (i) diagnosis of Bipolar I or Bipolar II disorder as determined per the Structured Clinical Interview for the DSM-IV (First et al. 2002); (ii) ages 18–65; (iii) premorbid IQ greater than or equal to 70 as estimated by the reading subtest of the Wide Range Achievement Test (Wilkinson & Robertson 2006); (iv) ability to provide informed consent; (v) fluency in English per self-report from the participant; (vi) current remission of depressive symptomatology as indicated by a score of 8 or less on the Bipolar Depression Rating Scale (Berk et al. 2007); (vii) current remission of manic symptoms as indicated by a score of 7 or less on the Young Mania Scale (Young et al. 1978); and (vii) agreement to use at least one form of birth control during study participation. Exclusion criteria included: (i) hypersensitivity to previous receipt of d-cycloserine per participant report; (ii) epilepsy or history of seizures; (iii) meeting DSM-IV criteria for alcohol or drug abuse in the past month or dependence in the past three months; (iv) active suicidal or homicidal ideation; (v) initiation or increase in dosage of any antidepressant within six weeks or mood stabilizer within four weeks; (vi) previous or current participation in cognitive remediation; (vii) current use of d-cycloserine; (viii) reduced kidney or liver functioning, B12 deficiency, folic acid deficiency, megaloblastic anemia; or sideroblastic anemia; (ix) current use of any medication known to have problematic interactions with d-cycloserine, including etionamide and isoniazid; (x) history of the blood disease porphyria; (xi) current active symptoms of psychosis defined as not meeting remission criteria for psychotic symptoms (Andreasen et al. 2005) using the Positive and Negative Syndrome Scale (Kay et al. 1987); (xii) evidence of dementia or other organic impairment that may reduce cognitive functioning; and (xiii) breastfeeding or pregnancy in female participants.

### Interventions

#### Cognitive remediation

For the current study, we will provide participants metacognitive remediation (MCR: (Breitborde et al. 2014)). As part of MCR, participants complete computerized cognitive training activities that are included in the program PSSCogRehab (Bracy 1995)--a computerized cognitive training program frequently used in past studies of cognitive remediation in serious mental illness (Kurtz et al. 2007; Greig et al. 2007; Bell et al. 2001; Fiszdon et al. 2004; Fiszdon et al. 2005; Fiszdon et al. 2006; Hogarty et al. 2004; Eack et al. 2009; Eack et al. 2007; Breitborde et al. 2011). This program provides participants with training in 4 areas of cognitive functioning: attention, visual-spatial abilities, memory, and problem-solving abilities. Participants initially complete simple training tasks in each domain and, once mastered, gradually progress to more difficult tasks.

After each PSSCogRehab task, participants complete a “metacognitive discussion” with a therapist designed to increase participants’ metacognitive skills. Outside of the psychiatric literature, improvements in metacognitive skills have been shown to facilitate improvements in cognitive performance and successful transfer of this knowledge across domains of functioning (Salomon & Perkins 1989; Perkins & Salomon 1994; Veenman et al. 2006; Schraw 1998).

Drawing on seminal work by Schraw and colleagues (Schraw 1998; Schraw & Dennison 1994; Schraw & Moshman 1995; Schraw et al. 2006), we conceptualize metacognition as comprised of two components: (i) knowledge about cognition and (ii) regulation of cognition. Knowledge about cognition refers to the ability to reflect on cognition and learning, whereas regulation of cognition refers to being able to control and regulate aspects of cognition and learning. During the metacognitive discussion, the therapist and participant review the participants’ (i) knowledge about cognition and (ii) regulation of cognition during the completion of PSSCogRehab task. With regard to the former, this discussion may include evaluating the pros and cons of different strategies to successfully complete a PSSCogRehab task. With regard to the latter, this discussion may include identifying strategies that individuals could use to reduce the negative effect of defeatist beliefs on their cognitive performance. At the end of the metacognitive discussion, the therapist and participant explore the possible “real-world” application of the metacognitive skills utilized in completing the PSSCogRehab activities. For example, the therapist and participant may (i) identify “real-world” situations where defeatist beliefs also reduce the participant’s cognitive performance and (ii) explore whether the participant could utilize the same strategies to cope with defeatist beliefs identified and practiced during the MCR session in these “real world” situations.

For the current study, participants will complete two, one-hour cognitive remediation sessions per week for 6 months (i.e., 26 weeks; 52 visits). In a previous study of MCR among individuals with first-episode psychosis (Breitborde et al. 2014), we found that individuals who received this dosage of MCR experienced greater cognitive and functional improvements (e.g., increased educational/occupational functioning) than individuals who completed only the PSSCogRehab computer activities (i.e., no metacognitive discussion).

#### d-Cycloserine vs. Placebo

Following the baseline evaluation, participants will present for twice weekly sessions where they will receive CR. Upon arrival at each visit, participants will be provided with a 250 mg capsule of d-cycloserine or placebo to ingest. To reduce the time needed for plasma levels of d-cycloserine to reach their peak concentration, participants will be asked not to eat any food for 12 hours prior to each visit (Zhu et al. 2001). Participants will also be required to wait one hour after ingesting the capsule to allow for the d-cycloserine to reach peak plasma levels (Zhu et al. 2001). After this one-hour period, participants will complete of one hour of CR.

### Randomization and treatment allocation

Treatment allocation of this study is depicted in Figure 1. Upon enrollment in the study, participants will be randomized using a 1:1 ratio to one of two study arms: (i) CR + DCS or (ii) CR + placebo. Randomization will be completed using a block randomization procedure with blocks of varying size. Both participants and study personnel will be blind with regard to participant assignment to either DCS or pharmacological placebo.

### Assessment battery

All participants will complete a number of assessments during study completion. Although the majority of measures will be completed twice over the course of the study (i.e., baseline and 6-month follow-up), certain measures will be administered on a more frequent basis. Study measures and administration schedule are summarized below and in Figure 1.

All assessors will complete a training program prior to administering study-related assessments. For assessments that require clinical judgment (e.g., symptom severity measures), assessors will rate a series of training videos prior to administration of these measures. Assessors will be required to reach specific reliability criteria (e.g., intraclass correlation ≥ 0.75 for ratings of continuous variables) as compared to expert ratings of these training videos.

### Primary outcome measure: change in global cognitive functioning

Cognitive functioning among study participants will be assessed at baseline and 6-month follow-up using the MATRICS Consensus Cognitive Battery (MCCB: (Nuechterlein et al. 2008)). For this study, the primary outcome measure will be change in the MCCB global cognition composite score from the baseline to 6-month assessment.

### Secondary outcome measures

#### Cognitive functioning: change in specific cognitive domains

The MCCB will also be used to assess change in specific domains of cognitive functioning among study participants. The MCCB measures seven specific domains of cognitive functioning: (i) processing speed; (ii) attention/vigilance; (iii) working memory; (iv) visual learning; (v) verbal learning; (vi) reasoning/problem-solving; and (vii) social cognition. Per existing recommendations (Yatham et al. 2010), the MCCB will be supplemented with the following measures to increase the appropriateness of this assessment battery to the specific cognitive deficits common among individuals with bipolar disorder: (i) California Verbal Learning Test (CVLT: (Ressler et al. 2004)); (ii) Stroop Test (Stroop 1935); (iii) Trail Making Test-Part B (Reitan & Wolfson 1985); and (iv) Wisconsin Card Sorting Test (WCST: (Heaton et al. 1993)). Finally, participants will also complete the Cognitive Neuroscience Test Reliability and Clinical Applications for Schizophrenia battery (CNTRACS: (Barch et al. 2012; Henderson et al. 2012; Ragland et al. 2012; Silverstein et al. 2012)) at both the baseline and 6-month assessment.

#### Cognitive functioning: rate of improvement

Recent evidence raises the possibility that DCS may not increase the benefit received from participation in psychosocial interventions, but instead may help individuals achieve this benefit more quickly (Siegmund et al. 2011; Kushner et al. 2007; Wilhelm et al. 2008; Chasson et al. 2010). Thus, to assess the rate of improvement in cognitive functioning, participants will complete the Repeatable Battery for the Assessment of Neuropsychological Status (RBANS: (Randolph 1998)). This brief assessment battery measures five domains of cognitive functioning: (i) immediate memory; (ii) visuospatial and constructional skills; (iii) language; (iv) attention; and (v) delayed memory. With four alternative forms, the RBANS is designed specifically to reduce the practice effect associated with multiple administrations of a neuropsychological test. This assessment battery will be administered at baseline, Week 5, Week 14, and 6-month follow-up.

#### Cognitive functioning: subjective assessment

The Measure of Insight into Cognition-Self Report (MIC-SR: (Medalia et al. 2008)) is a 12 item questionnaire designed to assess self-perception of cognitive abilities. This measure assesses subjective perceptions of three domains of cognitive functioning: (i) attention; (ii) memory; and (iii) executive functioning. Although a clinician-rated version of Measure of Insight into Cognition (MIC-CR: (Medalia & Thysen 2008)) is available, we opted not to include this measure as it assesses individuals’ awareness of their cognitive deficits as opposed to their subjective perception of the severity of their cognitive deficits (Saperstein et al. 2012).

#### Medication use, adherence, and side effects

Several psychiatric medications commonly used in the treatment of bipolar disorder may influence glutamate signaling (e.g., lamotrigine and lithium (Sitges et al. 2007; Dixon & Hokin 1998)). As such, medication use among study participants will be tracked using a semi-structured assessment used in past studies of cardiovascular functioning in first-episode psychosis (Srihari et al. 2013; Phutane et al. 2011). Adherence to prescribed psychiatric medication not including DCS will be assessed at baseline and 6-month follow-up using the Medication Adherence Rating Scale (MARS: (Thompson et al. 2000)). The Systematic Assessment For Treatment Emergent Events (SAFTEE: (Levine & Schooler 1986)) will be administered at each cognitive remediation visit to assess for side-effects associated with DCS.

#### Metacognition

The Metacognitive Awareness Inventory (MAI: (Schraw & Dennison 1994)) is 52-item questionnaire designed to measure metacognitive abilities (i.e., the ability to evaluate, regulate, and understand learning and cognitive skills). The MAI is designed to assess the two domains of functioning hypothesized to comprise metacognitive abilities: (i) knowledge about cognition and (ii) regulation of cognition (Brown 1987; Flavell 1987). Knowledge about cognition refers to the ability to reflect on cognition and learning, whereas regulation of cognition refers to being able to control and regulate aspects of cognition and learning.

#### Personality traits

The Ten Item Personality Inventory (TIPI: (Gosling et al. 2003)) will be used to assess the Big Five Personality Traits among study participants: extraversion, agreeableness, conscientiousness, emotional stability, and openness to experience. This measure has been shown to have good discriminant and convergent validity when compared to other longer assessments of the Big Five Personality Traits (e.g., NEO Personality Inventory (Costa 1992)).

#### Quality of life

Participants’ quality of life will be assessed using the WHO Brief Quality of Life scale (WHOQOL-BREF: (Group 1998)). This 26-item self-report measure assesses four domains of quality of life: (i) physical health; (ii) psychological health; (iii) social relationships; and (iv) quality of the environment. Additionally, all participants will complete a more specific measure of health-related quality of life (i.e., the RAND 36-Item Health Survey: (Hays et al. 1998)). This questionnaire measures 8 domains of health-related quality of life: (i) physical functioning; (ii) pain; (iii) role limitations due to physical health; (iv) role limitations due to emotional health; (v) energy and fatigue; (vi) social functioning; (vii) emotional well-being; and (viii) general health.

#### Real world functioning

The University of California, San Diego, Performance-Based Skills Assessment-Brief (UPSA-B: (Mausbach et al. 2007)) will be used to assess real world functioning among study participants (i.e., communication and financial skills). This measure is highly correlated with the full UPSA (*r* = 0.91) and predicts residential independence among individuals with severe mental illnesses (Mausbach et al. 2007).

#### Recovery

Participants’ stage of recovery in bipolar disorder will be assessed using the Stages of Recovery Instrument (STORI: (Andresen et al. 2006)). Drawing on Andresen and colleagues’ model of recovery in severe mental illness (Andresen et al. 2003), this 50-item questionnaire assigns individuals to one of five stages of recovery: (i) moratorium (i.e., a period of loss and hopelessness); (ii) awareness (i.e., recognition that one can achieve a fulfilling life and positive sense of self); (iii) preparation (i.e., developing skills needed to achieve desired fulfilling life and positive sense of self); (iv) rebuilding (i.e., actively working to achieve fulfilling life and positive sense of self); and (v) growth (i.e., maintaining a fulfilling life and positive sense of self).

#### Social functioning

The Social Functioning Scale (SFS: (Birchwood et al. 1990)) will be used to measure level of social functioning among study participants. The SFS is a 79-item instrument that assesses seven areas of functioning: (i) social engagement/withdrawal; (ii) interpersonal behavior/communication; (iii) participation in prosocial activities; (iv) participation in recreational activities; (v) independence competence (i.e., ability to perform tasks of independent living); (vi) independence performance (i.e., completion of tasks of independent living); and (vii) educational/vocational functioning.

#### Service utilization

Participants’ use of healthcare services will be assessed using the Service Use and Resource Form (SURF: (Rosenheck et al. 2003)). This clinician-administered measure assesses the frequency of participants’ use of inpatient and outpatient psychiatric and medical services over the past six months. The SURF also tracks participants’ level of contact with the legal system, insurance status, and financial resources.

#### Symptomatology

Severity of bipolar symptomatology will be assessed at each CR visit using both clinician-administered and self-report scales. Clinician-administered scales include the Inventory of Depressive Symptomatology (Rush et al. 2006), Bipolar Depression Rating Scale (Berk et al. 2007), and Young Mania Scale (Young et al. 1978). Self-report scales include the Inventory of Depressive Symptomatology Self-Report Scale (Rush et al. 2006) and the Altman Self-Rating Mania Scale (Altman et al. 1997).

#### Treatment motivation and therapeutic alliance for CR/Active control condition

Following the completion of each cognitive remediation or active control visit, participants will complete the Intrinsic Motivation Inventory (Choi et al. 2010) and the Session Rating Scale (Duncan et al. 2003) to assess their motivation to participate in CR and their alliance with the clinician delivering the intervention, respectively.

### Proposed analyses

Prior to the analyses, data will be screened for outliers and departures from a normal distribution. Data analyses will be completed using an “intention-to-treat” principle (Montori & Guyatt 2001). Consequently, data from all participants will be included in the analyses regardless of their level of participation in study interventions over the course of the project. Per existing statistical guidelines (Collins et al. 2001; Graham 2009), missing data will be estimated using multiple imputation (Rubin 1987).

The interaction between time (i.e., baseline vs. 6-month) and treatment condition (DCS vs. placebo) on our primary outcome variable (MCCB composite cognition score) will be assessed using a repeated measures ANOVA. An a priori estimate of statistical power was completed using G*Power 3.1 (Faul et al. 2009). Assuming a correlation greater than or equal to 0.40 between baseline and six month MCCB composite cognition scores and a medium effect size (i.e., *f* = 0.25), the power to detect a statistically significant interaction between time and treatment condition (i.e., cycloserine/placebo) is greater than or equal to 0.80. Should we find a statistically significant time X treatment condition interaction, post-hoc probing of the interaction will be completed using t-tests with Bonferroni corrections to maintain an alpha of 0.05.

## Discussion

Cognitive deficits are a rate-limiting factor in functional recovery among individuals with bipolar disorder (Bearden et al. 2011; Baune et al. 2013; Dickerson et al. 2004). Unfortunately, treatment options for these deficits are limited. The results of the proposed study may reveal a valuable intervention strategy (i.e., CR with concurrent DCS) to improve cognitive functioning among individuals with bipolar disorder. Ultimately, this treatment strategy may prove useful in addressing the cognitive deficits that are ubiquitous across serious mental illnesses.
